# Assessing the Level of Awareness About Breath-Holding Spells Among the General Population in the Makkah Region, Saudi Arabia

**DOI:** 10.7759/cureus.50659

**Published:** 2023-12-17

**Authors:** Mohammed Abuaish, Heba Y Dosh, Khulud A Boubsit, Sarah A Munshi, Suhaib Y Dosh, Sumaya Z Khayat, Mohammed A Alharthi, Elaf F Alharbi

**Affiliations:** 1 Pediatric Emergency Medicine, Department of Pediatrics, Faculty of Medicine, Umm Al-Qura University, Makkah, SAU; 2 Medicine, College of Medicine, Umm Al-Qura University, Makkah, SAU; 3 Medicine, Ibn Sina National College for Medical Studies, Jeddah, SAU; 4 Pediatrics, Maternity Children Hospital, Makkah, SAU; 5 Pediatric Emergency, Maternity Children Hospital, Makkah, SAU

**Keywords:** makkah, children, bhs, breath-holding spells, awareness

## Abstract

Background and objective

Breath-holding spell (BHS) is a serious condition that affects healthy and normal children. It is a paroxysmal non-epileptic disorder and usually occurs after the child gets angry, annoyed, or aroused. In such a scenario, an episode of crying and silent expiration associated with color changes, either cyanosis or pallor, leads to loss of consciousness in the child. In Saudi Arabia, studies assessing the awareness among parents about BHS have been scarce. In light of this, this study aimed to evaluate the awareness of BHS among parents in Makkah, Saudi Arabia, in 2023.

Methodology

This was a cross-sectional study conducted between June and October 2023 by using Google Forms to collect data about awareness of risk factors of BHS among the general population in Makkah. We employed a validated and self-generated survey that was distributed through social media platforms.

Results

The study included 602 participants; 138 (22.9%) of them had witnessed BHS episodes. Of note, 407 (67.6%) thought that there was a connection between BHS and iron deficiency anemia. A significant majority (n=565, 93.9%) mentioned that spells can lead to passing out and seizures, and 542 (90%) thought that spells are dangerous. The majority (n=479, 79.6%) reported that the actions required during spells involve laying the child on the floor, keeping the child away from anything sharp, staying with the child, and calling 911 if the child remains blue or is not breathing for longer than a minute. The most common source of information for the participants was the Ministry of Health (n=182, 30.2%).

Conclusions

This study found a generally low level of awareness among parents regarding several aspects of BHS. Hence, we recommend conducting educational campaigns to ensure parents have accurate information about BHS so that they can respond appropriately to its occurrence in children.

## Introduction

Breath-holding spell (BHS) is an alarming and common incident that affects healthy and normal children. It is a paroxysmal non-epileptic disorder [1,2]. It usually happens after the child gets angry, annoyed, or aroused and is characterized by an episode of crying and silent expiration associated with color changes, either cyanosis or pallor, which leads to loss of consciousness [3,4]. BHS frequently starts during the first year of life, and most children outgrow these spells by the age of six years [4]. It can be clinically classified into two types according to the color change the child manifests during the episode: cyanotic (blue child) and pallid (pale child); in rare cases, both types can occur in the same child (mixed type) [1,4]. The mechanism behind BHS is complicated, not wholly understood, and most likely a multifactorial phenomenon. Iron and zinc deficiency have been implicated as causes of BHS, based on the positive responses of some cases to iron therapy. One of the important risk factors is autonomic dysregulation, which alters heart function while decreasing cerebral blood flow [4]. While this condition affects both males and females equally, a slight male predominance has been observed. The family history is positive in 20-35% of cases [5].

Hüdaoglu et al. have reported that family structure and the mothers’ attitudes do not appear to influence the development of BHS [6]. In contrast, Kursat et al. have stated that the lower educational status of mothers is a risk factor for BHS. This condition may result in improper feeding habits throughout the first year of life, resulting in deficiencies, especially of iron, which is one of the major causes of BHS. It has also been revealed that the birth sequence and the father's educational status influence the occurrence of BHS [7]. Therefore, household size, paternal education, and a family history of mental illness are statistically significant predictors of BHS in children under the age of five years [8].

In Saudi Arabia, studies that assess the awareness among parents about BHS have been scarce, and hence there is a need for more research to cover this gap. Therefore, this study aimed to evaluate the awareness of BHS among parents in Makkah, Saudi Arabia, in 2023.

## Materials and methods

This was a descriptive cross-sectional study involving a Google Form-based survey [9] to collect data about awareness of risk factors for BHS among the general population in the Makkah region between June and October 2023. The inclusion criteria were adults of both genders aged 18 years or above living in Makkah. The exclusion criteria included healthcare providers, as their awareness level is not representative of the general population. The survey was distributed through social media platforms (Twitter, WhatsApp, Facebook, and email), and participants were selected via a convenient sampling technique and snowball method. The study did not include any dropped-out participants, as the nature of our study design did not include follow-up. 

To calculate the required minimum sample size, the population of Makkah City was taken into account (8,021,463, based on 2023 national statistics [10]); keeping the confidence interval at 95% and anticipated % frequency as 50%, the sample size was determined to be 385 individuals. The OpenEpi website was used for the calculation [11].

The survey (see Appendix) format was created by the authors due to the limited availability of similar validated tools in the literature. Thus, for validation purposes, an assessment was conducted by four content experts at Umm Al-Qura University (UQU) who specialized in pediatric health, pulmonology, and epidemiology. In addition, a medical educationist assessed the survey's face validity and found that it was fit to accomplish the objectives and that the flow of the questions was in a logical sequence. To assess the questionnaire's reliability, a pilot study using the newly developed questionnaire was conducted involving 30 participants; the questionnaire was then modified according to the respondents' feedback and their answers were excluded from the final analysis. The survey was distributed in both English and Arabic (the mother tongue of the participants) languages. Subsequently, all the data were translated back into English for analysis and data presentation.

The ethical approval was obtained from the Biomedical Research Ethics Committee of UQU (approval no: HAPO-02-K-012-2023-06-1677) and no data were collected before ethical approval. We outlined the study participants' responses, and frequencies and percentages were calculated to quantify all categorical variables. Statistical analyses of data were performed using IBM SPSS Statistics [12] version 27.0.1 (IBM Corp., Armonk, NY).

## Results

Table 1 provides a comprehensive overview of the demographic composition of the parents. Regarding gender, the study revealed that 188 (31.2%) of the participants were males, while the majority, constituting 414 (68.8%) participants, were females. The highest number of participants was between the age group of 18-24 years (218, 36.2%), and 118 participants (19.6%) were aged between 25-34 years. Regarding marital status, 280 of the participants (46.5%) were married, 17 (2.8%) were divorced, six (1.0%) were widowed, and 299 (49.7%) were singles. More than half of the participants had a postgraduate education (409, 67.9%) while 193 had an undergraduate education (32%). As reported by the participants, 138 (22.9%) of them had witnessed BHS, while the remaining 464 participants (77.1%) never had. Regarding the children of the participants, half of them were males (50.0%), with a mean age of 15 months.

**Table 1:** Demographic characteristics of the parents (n=602)

Characteristics	N (%)
Gender	
Male	188 (31.2%)
Female	414 (68.8%)
Age, years	
Less than 18	27 (4.5%)
18–24	218 (36.2%)
25–34	118 (19.6%)
35–44	96 (15.9%)
45–54	80 (13.3%)
More than 55	63 (10.5%)
Marital status	
Married	280 (46.5%)
Divorced	17 (2.8%)
Widowed	6 (1.0%)
Single	299 (49.7%)
Educational level	
Postgraduate	409 (67.9%)
Undergraduate	193 (32.1%)
Did you witness a breath-holding spell before?	
Yes	138 (22.9%)
No	464 (77.1%)

Table 2 illustrates participants' knowledge about BHS; 195 (32.4%) of the respondents stated that a BHS is when a child holds their breath, while 407 (67.6%) responded "True" to that statement. Similarly, 47 participants (7.8%) answered "False" to the statement that breath-holding leads to the child passing out, while the remaining 555 (92.2%) recognized this as "True." About the connection between BHS and iron deficiency anemia, 195 participants (32.4%) believed it to be "False," while 407 (67.6%) recognized it as "True." Regarding cyanotic and pallid BHS, 10.3% and 28.6%, respectively, identified them as "True." The majority (565, 93.9%) recognized that spells can lead to seizures ("True"). The danger of the spells was recognized by 542 (90.0%) ("True"), and 529 (87.9%) admitted that children have no control over the spells.

**Table 2:** Knowledge about breath-holding spells in the cohort

Question	False, n (%)	True, n (%)
Breath-holding spell is when a child holds their breath after a certain trigger (anger, pain, frustration)	195 (32.4%)	407 (67.6%)
Breath-holding leads to the child passing out (syncope or loss of consciousness)	47 (7.8%)	555 (92.2%)
Breath-holding spells are involuntary	73 (12.1%)	529 (87.9%)
Spells could be followed by seizures	37 (6.1%)	565 (93.9%)
Breath-holding spells are dangerous for children	60 (10.0%)	542 (90.0%)
Breath-holding spells are more common in children with Iron deficiency anemia	195 (32.4%)	407 (67.6%)

A notable proportion (477, 79.2%) acknowledged that these spells could happen when the child is "Angry," "Frustrated," and due to "Pain," while 88 (14.5%), 6 (1.0%), and 32 (5.3%), respectively, indicated these specific triggers as sole triggers. The majority (479, 79.6%) recognized that in the event of BHS, it is advisable to perform various actions: "Lay your child in the crib or on the floor," "Keep your child away from anything hard or sharp," "Stay with your child," and "Call 911 if your child remains blue or is not breathing for longer than a minute." As for the sources of information, the Ministry of Health was cited by 182 (30.2%), Scientific Journals by 82 (13.6%), TV Programs by 58 (9.6%), and 377 (62.6%) relied on "Family - Close Friends."; 123 (20.4%) stated that they consulted a doctor, while "Social Media" (0, 0.0%) and "Others" 38 (6.3%) had a lower reported influence.

## Discussion

This study aimed to assess parents' awareness of BHS in the Makkah region; our decision to conduct this study was primarily spurred by the inadequate number of published studies on the topic and the significance of parental knowledge regarding such an episode, given its frightening nature and high prevalence in children. In this study, which included 602 participants, 470 participants reported witnessing at least one BHS incident before; however, we need more data regarding the occurrence of these spells in Saudi Arabia to make a correlation. Half of the children were males, with a mean age of 15 months, which is consistent with the age of onset reported in the literature, which is typically between 6 and 18 months [8].

Most of the participants in our study answered almost all of the questions about the knowledge of BHS correctly, with a few exceptions. Regarding the statement "Despite the dramatic nature of the symptoms, the spells are dangerous," the majority (542, 90.0%) of subjects chose the option "Accurate," while the correct answer was "False." Also, regarding the statement "Children are not intentionally holding their breath and have no control over the spells," most participants chose "accurate", while the correct answer was "False." This led us to conclude that these spells are misunderstood in terms of their prognosis and that people are unaware of their benign nature. Several studies have observed and reported that despite the benign nature of BHS [13], parents feel quite distressed about it [14].

On the other hand, nearly all of the subjects were aware of what BHS is, that a child can lose consciousness during one, and that children with iron deficiency anemia are more likely to experience one. Additionally, a substantial number of parents were aware of the definitions of the BHS, as well as pallid and cyanotic spell subtypes. Roughly all of the participants were aware that anger, pain, and frustration could provoke spells. They also demonstrated knowledge about the proper course of action to take when children experience one, which is to lay them down on the floor or in their crib, keep them away from anything hard or sharp, stay with them, and call 911 if they stay blue or stop breathing for more than a minute.

Family and close friends were the primary sources of knowledge for most of the participants, followed by the Ministry of Health, doctor consultations, scientific journals, and TV programs, respectively. We need to implement additional awareness campaigns through social media platforms because none of the participants chose social media as a source of information. The effects of awareness campaigns on parents in Saudi Arabia are known to have a remarkable impact [15]. A study conducted in Turkey in 2013 compared the prevalence of BHS in different studies conducted in various countries and periods. It mentions risk factors such as consanguinity and mothers' lower education status, as well as the role of genetic inheritance. It also discusses the impact of birth sequence and fathers' education status on the prevalence of BHS. It concludes by mentioning the typical age of onset for these spells and how it aligns with the literature [7].

While our study focuses more on the findings and knowledge of participants, the above-mentioned study provides a broader perspective by comparing findings from different studies and highlighting additional risk factors and factors affecting the prevalence of BHS. Another study conducted in Turkey in 2004 provided more general information about BHS. It included 30 mothers of children with BHS and involved a comparison with a group comprising the same number of mothers of healthy children. Their instrument scales were employed in the clinic, and the study mentions that these spells occur in around 5% of healthy children, with male children experiencing them at an earlier age than females. The disease often disappears by the age of four years and rarely occurs after the age of six years. Iron deficiency anemia is frequently observed in children with BHS, and iron therapy has been shown to be effective in reducing the frequency of the spells. It also mentions a possible association between elevated vagal response and autonomic dysregulation in these children. It mentions a potential genetic causative factor and suggests that attitude problems in both mother and child may trigger spells [6].

Generally, our study focuses on assessing parents' awareness of BHS, while the aforementioned study provides more general information about these spells, including prevalence, age of onset, potential causes, and treatment options. Another study, conducted at Fitzsimons General Hospital in Denver, Colorado, involved 697 children and collected their data by interviewing their mothers. It found that BHS in children is a common phenomenon that can range from mild to severe. The study found that mild BHS, characterized by prolonged breath-holding and cyanosis, had a prevalence of 4.7%. Severe BHS, which progressed to unconsciousness or convulsions, had a prevalence of 1.7%. The study also explored the relationship between BHS and emotional factors, such as parent-child conflicts. It was suggested that therapy should focus on easing these conflicts [16]. Compared to our study, both these studies emphasize the importance of understanding and addressing BHS, but they focus on different aspects. Our study primarily focuses on parental awareness and education, while the study from Denver focuses on the frequency, severity, and emotional factors associated with BHS in children.

Our study provides unique insights into the awareness of BHS in the Makkah population, which is a major holy city with a high rate of international visitors for Hajj and other pilgrimages. However, it has several limitations. One of these pertains to the fact that we employed a convenient sampling technique and conducted the study on an online platform, which may limit the generalizability of our findings. Additionally, an element of reporting bias may have crept in since the study relied on self-reported information that might be influenced by participants' opinions and ability to understand the items or their proclivity to express their feelings in a certain manner. Lastly, we used a cross-sectional design, which precluded the ability to draw causal conclusions.

## Conclusions

This study found a significantly low level of awareness regarding several aspects of BHS among parents in Makkah, Saudi Arabia. While most participants were aware of the general concept of BHS, they still lacked awareness regarding the dangerous nature of the spells. Hence, we recommend that further educational campaigns be conducted to ensure parents have accurate information and can respond appropriately to BHS in children. Awareness campaigns are generally known to have a remarkable impact on parents in Saudi Arabia.

## Appendices

The questionnaire used in the study is presented in Table 3.

**Table 3:** Questionnaire used in the study

1. Introduction, consent, and inclusion criteria assessment
2. Gender
Male
Female
3. Age
<18 years
18–24 years
25–34 years
35–44 years
45–54 years
55 years and above
4. Marital status
Married
Divorced
Widowed
Single
5. Nationality
Saudi
Non-Saudi
6. Educational level
Primary to secondary school
Diplomatic
Bachelor's degree
Master's degree to doctorate
7. Did you witness breath-holding spells before?
Yes --> will go to Q8
No --> will go to Q19
8. Gender of child with BHS
Male
Female
9. Relationship
Son/daughter
Brother/sister
Other: ………..
10. Age: …………….. (specify months or years)
11. Weight (kg): ……………
12. Provocation factors
Anger
crying
Anger and crying
Others: ………..
13. Spell type
Cyanotic
Pallid
Mixed
14. Duration of spells in seconds: …………..
15. Frequency of spells
Only one time
Daily
More than one spell a day
Weekly
Monthly
16. Positive family history of BHS
Yes
No
17. Positive family history of seizures
Positive family history of seizures
Second degree relative
Family history of BHS
18. Associated symptoms
Loss of consciousness
Seizure-like movements
Others: ……………………
19. Breath-holding spell is when a child holds their breath
True
False
20. Breath-holding spell can happen after the child is
Angry
Frustrated
In pain
All of the above
21. Breath-holding leads to the child passing out
True
False
22. If the child’s face turns blue, it’s called a cyanotic breath-holding spell
True
False
23. If the child’s face turns white, it’s called a pallid breath-holding spell
True
False
24. Spells can make kids pass out for up to a minute and kids might have seizures
True
False
25. Despite the dramatic nature of the symptoms, the spells are dangerous
True
False
26. Children are not intentionally holding their breath and have no control over the spells
True
False
27. What should I do if my child has a breath-holding spell?
Lay your child in the crib or on the floor
Keep your child away from anything hard or sharp
Stay with your child
Call 911 if your child remains blue or is not breathing for longer than a minute
All of the above
28. What are your sources of information?
Ministry of Health
Scientific journals
Social media
TV programs
Family - close friend
Doctor consultation
Others: ………….

## References

[1] DiMario FJ Jr (1992). Breath-holding spells in childhood. Am J Dis Child.

[2] Leung AKC, Leung AAM, Wong AHC, Hon KL (2019). Breath-holding spells in pediatrics: a narrative review of the current evidence. Curr Pediatr Rev.

[3] Flodine TE, Shah M, Mendez MD (2023). Breath Holding Spells. https://www.ncbi.nlm.nih.gov/books/NBK539782/.

[4] Azab SF, Siam AG, Saleh SH (2015). Novel findings in breath-holding spells: a cross-sectional study. Medicine (Baltimore).

[5] Choudhury AM‏ (2020). Breath holding spell - a frightening event for parents. JMRKSH.

[6] Hüdaoglu O, Dirik E, Yiş U, Kurul S (2006). Parental attitude of mothers, iron deficiency anemia, and breath-holding spells. Pediatr Neurol.

[7] Carman KB, Ekici A, Yimenicioglu S, Arslantas D, Yakut A (2013). Breath holding spells: point prevalence and associated factors among Turkish children. Pediatr Int.

[8] Hesamifar M, Daryoushi H, Sedighi M (2021). Evaluation of the predictors of breath holding spell (BHS) in under 5-year-old children in Iran: a hospital-based case-control study. Int J Pediatr.

[9] (2023). Google Forms for survey. https://www.google.com/forms/about/.

[10] (2023). Saudi Census 2022 (by the General Authority for Statistics). https://portal.saudicensus.sa/portal/public/1/15/1367?type=DASHBOARD.

[11] (2023). Open Source Epidemiologic Statistics for Public Health (Open Epi). https://www.openepi.com/Menu/OE_Menu.htm.

[12] (2023). IBM SPSS Statistics. https://www.ibm.com/products/spss-statistics.

[13] Leung AC, Coenegrachts K (2011). Common Problems in Ambulatory Pediatrics: Anticipatory Guidance and Behavioral Pediatrics. Nova Science.

[14] Leung AK, Leung AA, Wong AH, Hon KL (2019). Breath-holding spells in pediatrics: a narrative review of the current evidence. Curr Pediatr Rev.

[15] Temsah MH, Aljamaan F, Alhaboob A (2022). Enhancing parental knowledge of childhood and adolescence safety: an interventional educational campaign. Medicine (Baltimore).

[16] Linder CW (1968). Breath-holding spells in children. Studies of frequency, severity, management. Clin Pediatr.

